# Exploiting the Combination of Natural and Genetically Engineered Resistance to Cassava Mosaic and Cassava Brown Streak Viruses Impacting Cassava Production in Africa

**DOI:** 10.1371/journal.pone.0045277

**Published:** 2012-09-25

**Authors:** Hervé Vanderschuren, Isabel Moreno, Ravi B. Anjanappa, Ima M. Zainuddin, Wilhelm Gruissem

**Affiliations:** Department of Biology, Plant Biotechnology, ETH Zurich, Zurich, Switzerland; Nanjing Agricultural University, China

## Abstract

Cassava brown streak disease (CBSD) and cassava mosaic disease (CMD) are currently two major viral diseases that severely reduce cassava production in large areas of Sub-Saharan Africa. Natural resistance has so far only been reported for CMD in cassava. CBSD is caused by two virus species, *Cassava brown streak virus* (CBSV) and *Ugandan cassava brown streak virus* (UCBSV). A sequence of the CBSV coat protein (CP) highly conserved between the two virus species was used to demonstrate that a CBSV-CP hairpin construct sufficed to generate immunity against both viral species in the cassava model cultivar (cv. 60444). Most of the transgenic lines showed high levels of resistance under increasing viral loads using a stringent top-grafting method of inoculation. No viral replication was observed in the resistant transgenic lines and they remained free of typical CBSD root symptoms 7 month post-infection. To generate transgenic cassava lines combining resistance to both CBSD and CMD the hairpin construct was transferred to a CMD-resistant farmer-preferred Nigerian landrace TME 7 (Oko-Iyawo). An adapted protocol allowed the efficient *Agrobacterium*-based transformation of TME 7 and the regeneration of transgenic lines with high levels of CBSV-CP hairpin-derived small RNAs. All transgenic TME 7 lines were immune to both CBSV and UCBSV infections. Further evaluation of the transgenic TME 7 lines revealed that CBSD resistance was maintained when plants were co-inoculated with *East African cassava mosaic virus* (EACMV), a geminivirus causing CMD. The innovative combination of natural and engineered virus resistance in farmer-preferred landraces will be particularly important to reducing the increasing impact of cassava viral diseases in Africa.

## Introduction

Cassava (*Manihot esculenta* Crantz) production in large areas of Africa is severely affected by two major viral diseases, cassava brown streak disease (CBSD) and cassava mosaic disease (CMD). CBSD is caused by two virus species, *Cassava brown streak virus* (CBSV) and *Ugandan cassava brown streak virus* (UCBSV) (picorna-like (+) ssRNA viruses, genus *Ipomovirus*, family *Potyviridae*) [1], [2]. CMD in Africa is caused by at least seven cassava mosaic geminivirus species (CMGs) [3]. Although, the etiologic agents, symptoms, and impact on cassava production are disease-specific, these two cassava viral diseases can overlap in their distribution [4], [5]. Despite evidence of synergism between the two viruses in *Nicotiana benthamiana* [6], field survey data do not support any interaction between the viruses [4]. However, it is notable that CBSD is particularly prevalent in CMD-resistant varieties [2], [5] that have been deployed in recent decades as part of CMD mitigation management strategies [7].

Because of the occurrence of CMD pandemics in Africa the cassava research community has traditionally focused its activities on CMD resistance. Breeding programs were conducted by several national programs and later at CGIAR centers to exploit natural CMD resistance using locally grown cultivars and a wild-relative of cassava (*Manihot glaziovii* Muell.-Arg.) [8], [9]. The molecular basis of natural resistance was investigated to generate molecular markers [10], [11] and ultimately to clone the dominant *CMD2* resistance gene. CMD resistance markers also allowed the introgression of the CMD resistance trait into farmer-preferred cassava cultivars [11]. Cassava landraces initially identified as CMD-resistant as well as CMD-resistant breeding lines were subsequently distributed to farmers in African regions where CMD was prevalent [7]. Contrary to CMD, limited natural resistance to CBSD, in a few cassava genotypes, has been identified and demonstrated for one of the viral species (UCBSV) causing CBSD [2]. The deployment of CMD-resistant cassava with different levels of susceptibility to viruses causing CBSD continues to impact production and alters the distribution and occurrence of UCBSV and CBSV in the field. Because CBSD resistance has not been found in cassava genotypes traditionally grown by farmers development of transgenic approaches are particularly relevant to reduce the increasing impact of CBSD in Africa.

**Figure 1:**
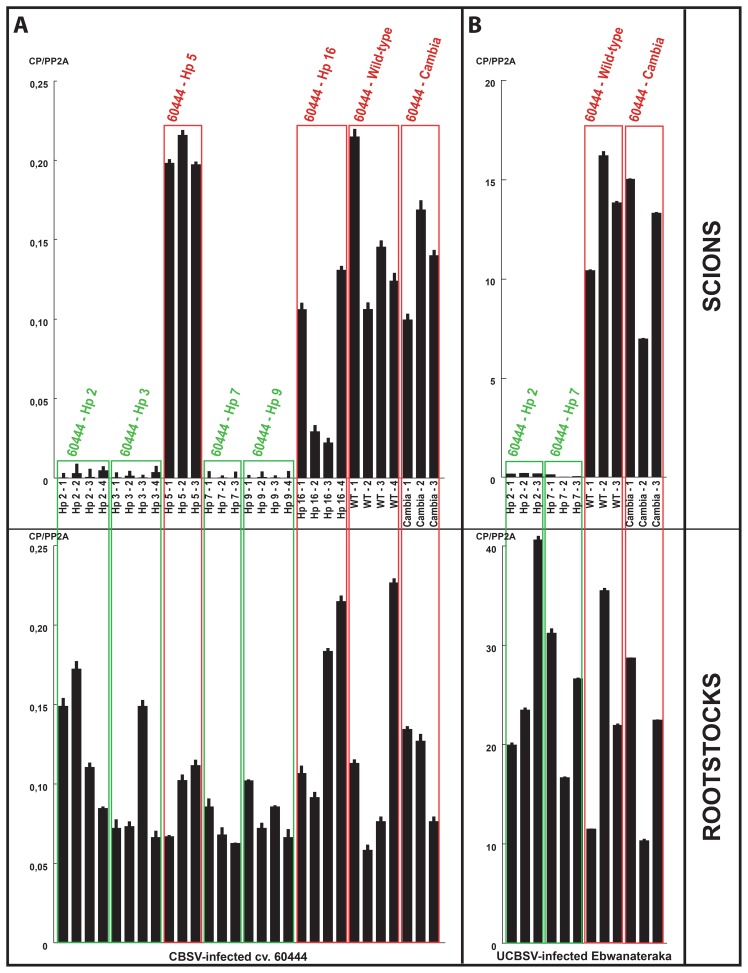
CBSV and UCBSV quantitation in rootstocks and cv. 60444 scions. A. CBSV quantitation by qPCR in CBSV-infected cv. 60444 roostock plants and corresponding grafted scions from transgenic, wild-type and control cv. 60444 lines. B. UCBSV quantitation by qPCR in UCBSV-infected Ebwanateraka roostock plants and corresponding grafted scions from transgenic, wild-type and control cv. 60444 lines. Numbers following the cassava line identifiers indicate the biological replicates.

Exploitation of the plant immune system against viruses [12] by expression of hairpin RNA homologous to viral sequences has proven effective to generate virus-resistant crops including cassava [13], [14], [15], [16]. The modularity of the hairpin RNA technology is suitable to generate plants resistant to multiple viral species. Stretches of viral sequences can be combined into a single hairpin construct [17]. Alternatively DNA sequences highly conserved between viral species can be targeted to generate transgenic plants with broad-spectrum resistance. Analysis of the CBSV and UCBSV isolates revealed high DNA sequence conservation in the 3′ region of the viral genome, where the gene for the coat protein (CP) sequence is located [18]. Here we show that stable resistance to both CBSV and UCBSV can be engineered by using hairpin RNA homologous to the 3′-end of the CBSV-CP sequence in the cassava model cultivar 60444 (previously referred to as TMS 60444). Using an improved cassava transformation protocol the technology was transferred to a selected Nigerian cassava landrace previously identified to be CMD-resistant [8], [19]. The combination of natural and engineered virus resistance is therefore a promising approach to combat multiple cassava viral diseases in Africa.

**Figure 2:**
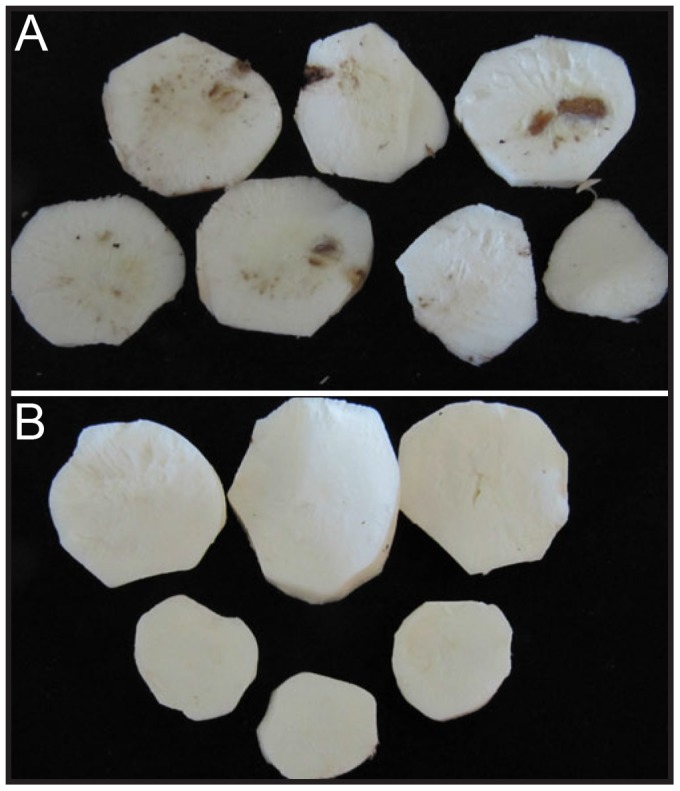
CBSV-associated symptoms in cassava storage roots. A. Storage roots of CBSV-inoculated cv. 60444 cassava at 7 month after stem propagation. B. Storage roots of CBSV-inoculated 60444–Hp 9 cassava line at 7 month after stem propagation.

**Table 1:** Phenotypic and molecular data of the 60444-Hp scion-propagated plants.

Scion identity	Virus species	Propagated cuttings	Symptomatic plants	Virus presence in scion(RT-qPCR)	Symptomatic roots
60444– Wild-type	CBSV	24	24	100%	45%
60444– Hp 2	CBSV	16	0	0%	0%
60444– Hp 3	CBSV	16	0	0%	0%
60444– Hp 7	CBSV	12	0	0%	0%
60444– Hp 9	CBSV	16	0	0%	0%

Stem cuttings from inoculated scions were propagated. Leaves and roots were evaluated 3 and 7 months after propagation, respectively.

## Materials and Methods

### Plant and Virus Material

*In vitro* culture of the cv. 60444 and the Nigerian landrace TME 7 (Oko-Iyawo) were used for cassava transformation. CBSV-[TAZ:DES:01] (GenBank JN091565.1) and UCBSV-[UG:Kab4∶07] (GenBank HM346952) isolates were initially propagated in AR34 and Ebwanateraka cassava cultivars, respectively. The CBSV isolate was transferred to cv. 60444 by grafting. CBSV-infected cv. 60444 scions were grafted onto EACMV-Ug (GenBank Z83257)-infected Ebwanateraka rootstocks. Upon development of typical CMD symptoms scions were subsequently multiplied to generate cv. 60444 rootstock plants co-infected with CBSV and EACMV-Ug. All cassava plants were grown under standard greenhouse conditions (27°C, 16 h light, 60% humidity).

### Plasmid Construction and Cassava Transformation

The CBSV coat protein (CP) sequence from position 538 to 1063 (GenBank JN091565.1) was amplified from CBSV-infected cassava following reverse transcription. Inverted sequences of the binary expression vector pRNAi-dsAC1 [14] were replaced by the amplified CBSV-CP fragment. The resulting construct was electroporated into *Agrobacterium tumefaciens* LBA4404.

cv. 60444 and TME 7 were transformed following an improved transformation protocol [20] with modifications for cassava TME landraces [21]. Fifty cotyledons were regenerated into plantlets and more than 90% of those rooted on selection media. Transgenic plantlets were subsequently selected for molecular characterization.

**Figure 3:**
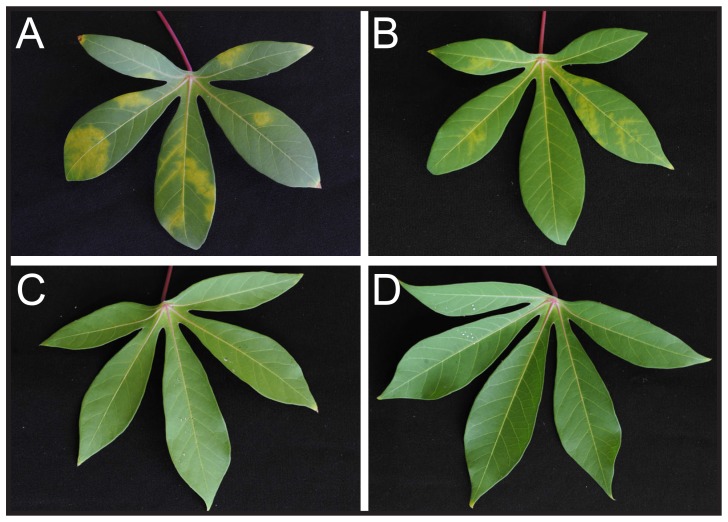
CBSV and UCBSV-associated symptoms in cassava leaves. A. Fully expanded leaf of wild-type TME 7 scion grafted on a CBSV-infected rootstock at 10 wpg. B. Fully expanded leaf of wild-type TME 7 scion grafted on an UCBSV-infected rootstock at 10 wpg. C. Fully expanded leaf of TME 7–Hp 9 scion grafted on a CBSV-infected rootstock at 10 wpg. D. Fully expanded leaf of TME 7 scion grafted on an UCBSV-infected rootstock at 10 wpg.

**Figure 4:**
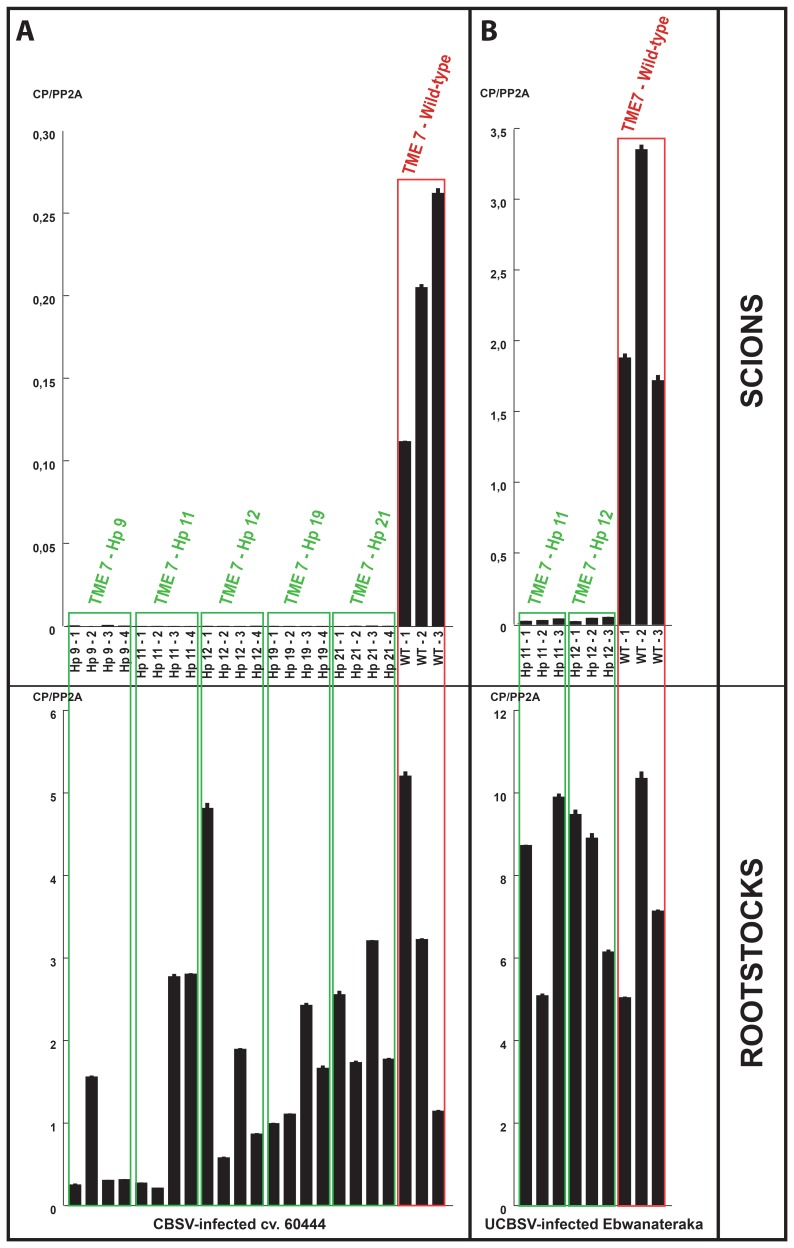
CBSV and UCBSV quantitation in rootstocks and TME 7 scions. A. CBSV quantitation by qPCR in CBSV-infected cv. 60444 roostock plants and corresponding grafted scions from transgenic, wild-type and control TME 7 lines. B. UCBSV quantitation by qPCR in UCBSV-infected Ebwanateraka roostock plants and corresponding grafted scions from transgenic, wild-type and control TME 7 lines. Numbers following the cassava line identifiers indicate the biological replicates.

### Viruses Alignment and Prediction of Small RNA-target Duplexes

CBSV and UCBSV sequences were aligned using the Clustal W method (DNASTAR Lasergene). A virtual repertoire of all possible 21nt small RNA sequences derived from the CBSV-CP (GenBank JN091565.1, 538–1063) hairpin was generated and used to predict targets on the UCBSV sequence (GenBank HM346952, 1034–1553) using RNAduplex (Vienna package) [22]. RNA duplexes with free energy below −25 kCal moI^−1^ were considered as potential target sites by CBSV-CP hairpin-derived small RNAs.

### Molecular Characterization of Transgenic Cassava Lines

Twenty µg of genomic DNA were digested with *Hind*III enzyme and blotted onto Hybond membrane (GE Healthcare LifeSciences). Membranes were hybridized with DIG-labeled (Roche Diagnostics) hptII probe amplified using *hptII* specific primers (*hptII-F:* 5′-TCTCGATGAGCTGATGCTTTGG and *hptII-R:* 5′-AGTACTTCTACACAGCCATCGG).

Small RNA detection from selected independent transgenic lines was performed according to a previously established protocol [23]. Small RNA were detected using a ^32^P-labelled oligonucleotide probe complementary to the CBSV-CP sequence (5′-ATCAGAATAGTGTGTCTGCTGG) and visualized using a phosphorimager (Bio-Rad Laboratories).

**Figure 5:**
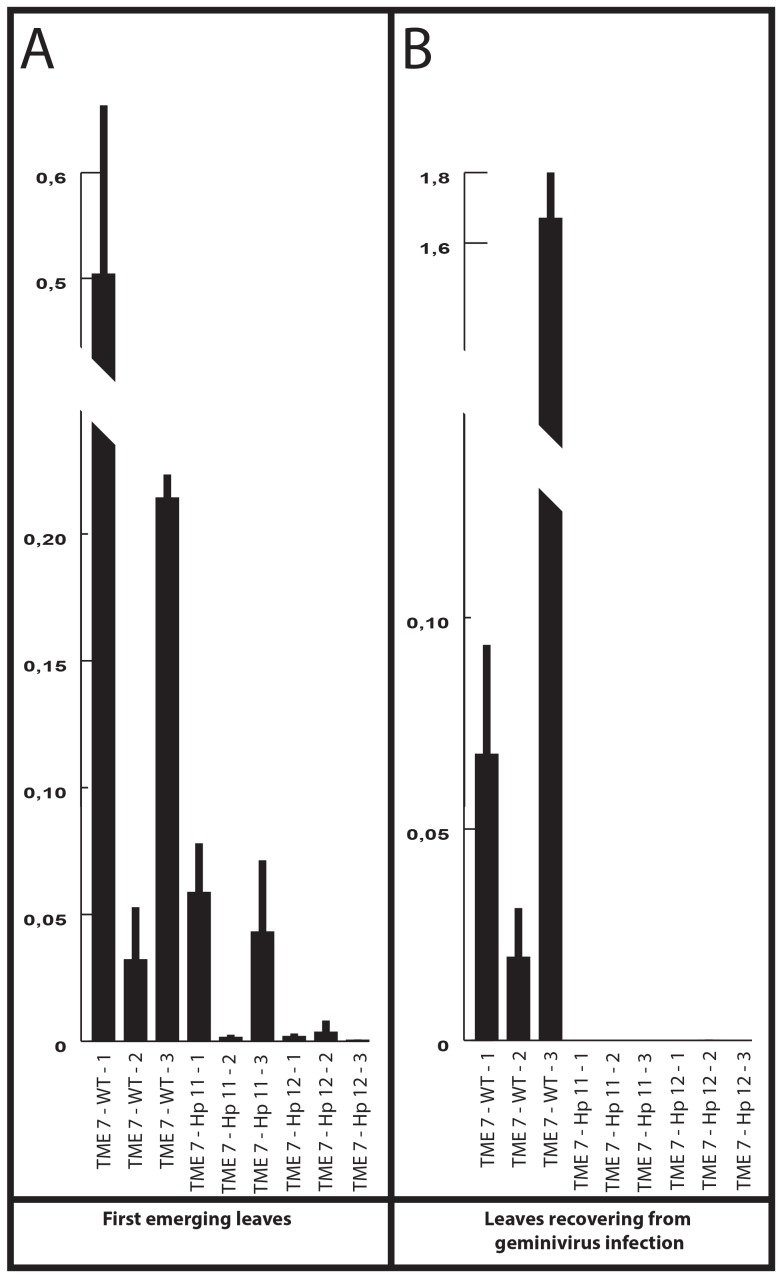
CBSV quantitation in TME 7 scions co-inoculated with CBSV and EACMV-Ug. CBSV quantitation by qPCR in transgenic and wild-type scions grafted onto cv. 60444 rootstocks co-inoculated with CBSV and EACMV-Ug at 8 wpg (A) and 14 wpg (B). Numbers following line identifiers indicate the biological replicates.

### Virus Inoculation and Resistance Evaluation

Scions from three-month old transgenic and wild-type cassava plants were grafted onto three-month-old wild-type rootstocks multiplied from virus-infected stock plants. EACMV-Ug and CBSV were co-inoculated in cassava by grafting. A standard top grafting procedure was used for cassava inoculation with CBSV [24]. Viral symptoms appearing on scions were monitored weekly and sampling of leaf material was performed at 8–14 weeks post-grafting (wpg). Scions were propagated at 14 wpg and viral symptoms were monitored for 7 months.

A standard leaf sampling procedure was performed on infected rootstocks and inoculated scions. In the standard sampling procedure three leaves below the point of grafting were sampled and pooled for RNA extraction. Similarly, three fully expanded leaves above the point of grafting were sampled and pooled to quantify viral load in the inoculated scions. Viral loads were quantitated by RT-qPCR based on a method for precise CBSV quantitation in cassava using the *PP2A* gene as internal control [24]. Primers specific for CBSV-CP sequence (*CBSV-CPF*: 5′-GAAGTTGAGAATTGGGCCATC and *CBSV-CPR*: 5′-CAGCTCTCCACGATTTCTCATT) and for UCBSV (*UCBSV-CPF*: 5′-CCATTTGAGGCTAGGAGATTGA and *UCBSV-CPR*: 5′-ACTTCCCCATCATCTGGTTCTC) were designed and validated for qPCR.

## Results

### Selection of Target Sequences for Effective CBSD Resistance Engineering

Genetic diversity has been best characterized for the CP sequence of CBSV and UCBSV [18]. Variability of the CP RNA sequences from CBSV and UCBSV isolates differs significantly between the N-proximal and the C-terminal regions. The N-proximal regions align poorly between isolates, while the C-proximal regions have retained sufficient conservation for alignment [2], [25]. Bioinformatics analysis of available CBSV and UCBSV complete genomes revealed that the C-proximal CP sequence ranks amongst the most conserved regions of the viral genome [18]. In our work a 525 base pair region was selected in the C-proximal CP sequence displaying 76.9% identity between UCBSV and CBSV isolates (Figure S1) to generate a hairpin construct. The selected sequence encompasses the CP-proximal region previously demonstrated by *in silico* analysis to produce the majority of the small interfering RNAs (siRNAs) with high similarity to both CBSV- and UCBSV-CP sequences [26]. Due to the higher virulence of CBSV isolates compared to UCBSV isolates [2] the CBSV C-proximal sequence was preferred over the UCBSV sequence for the production of the hairpin construct. We confirmed by *in silico* analysis that the UCBSV-CP sequence is a potential target of a large number of hairpin-derived small RNAs based on the free energy of siRNA-target duplexes (Figure S2, Table S1).

### C-proximal CBSV-CP Hairpin Construct Confers Resistance to CBSV in the Cassava Model Cultivar 60444

Most transgenic strategies have so far been evaluated in the model cultivar 60444 due to the difficulty of establishing robust and genotype-independent transformation of cassava [27]. We recently reported a protocol for routine and rapid production of transgenic cv. 60444 lines [20]. The protocol was used to generate over 50 transgenic cassava lines of which 18 were selected for molecular characterization (Figure S3). A subset of independent transgenic cassava lines with single construct insertions was selected for hairpin-derived small RNAs analysis. These transgenic cassava lines accumulated hairpin-derived small RNAs to different levels (Figure S4A). The 60444–Hp 9 line accumulated the highest level while four other transgenic lines (60444–Hp 2, 3, 7 and 16) displayed intermediate levels of small RNAs. CBSV-CP hairpin-derived small RNAs were not detectable in the line 60444–Hp 5. Following their molecular characterization, the transgenic lines were evaluated for CBSV resistance using a grafting method previously demonstrated to have a 100% transmission rate [24]. Scions from transgenic and control plants were grafted onto CBSV-infected cv. 60444 rootstocks. Wild-type and control scions as well as two transgenic lines displayed typical CBSD symptoms at 4 wpg. For reliable evaluation of resistance both rootstocks and scions were sampled and viral load quantitated at 10 wpg. Molecular quantitation of CBSV using qPCR confirmed the visual observations (Figure 1A, Table S2). CBSV was not detected in the four transgenic lines that were symptom-free in all replicates. The line 60444–Hp 5 that did not accumulate hairpin-derived small RNAs consistently accumulated a CBSV load comparable to wild-type. Despite CBSV-CP hairpin expression (Figure S4) all scions from the 60444–Hp 16 line allowed CBSV replication in the graft inoculation assay. The transgenic scions displayed typical CBSV symptoms, although viral load was reduced compared to wild-type (Figure 1A).

Using a quantitative method CBSV load was previously demonstrated to differ between cassava cultivars [24]. In our greenhouse conditions the cassava cultivar AR34 infected with CBSV displayed severe CBSD symptoms and high levels of CBSV replication compared to cv. 60444 and Ebwanateraka. In order to test the stability of the engineered resistance under high viral pressure selected CBSV-resistant transgenic lines were grafted onto CBSV-infected AR34 rootstock plants. Quantitation of the CBSV load in the rootstock plants confirmed the significantly higher CBSV accumulation in AR34 compared to cv. 60444 (Figure 1A and S4). Wild-type and control scions displayed typical CBSV symptoms at 4 wpg when grafted onto CBSV-infected AR34 rootstocks. CBSV quantitation at 10 wpg revealed high viral replication in the control and wild-type scions. None of the transgenic lines supported viral replication under high viral pressure (Figure S5) and all remained virus-free during the 4-month visual observation (Table S3).

### CBSV Resistance is Stable upon Multiplication of Inoculated Scions

Stem cuttings are used routinely by farmers to propagate cassava and they are also used to maintain virus isolates in greenhouses. In order to evaluate the stability of the engineered resistance and the capacity of CBSV to replicate in transgenic lines, CBSV-inoculated scions from transgenic lines and wild-type accessions were multiplied via stem cuttings. CBSV symptoms in the propagated stems were monitored for 7 months. Wild-type plants displayed typical CBSV symptoms on leaves and stems 4 weeks after planting. Transgenic scions remained symptom-free until harvest at 7 months after planting. CBSV detection and quantitation confirmed CBSV replication in all tested wild-type cuttings (Table 1). Storage roots of the 7-month-old plants were harvested and carefully sliced to assess the presence of corky necrosis in the starch-bearing tissues. Nearly half of the wild-type plants had CBSV-associated symptoms in their storage roots (Table 1, Figure 2). All harvested storage roots from transgenic CBSV-resistant lines were symptomless.

### C-proximal CBSV-CP Hairpin Construct Confers Resistance to UCBSV in the Cassava Model Cultivar 60444

Effective strategies to mitigate CBSD in the field require control of both virus species causing CBSD [28]. Despite lower pathogenicity [2], [26] UCBSV remains prevalent in several African regions. Therefore it was important to assess whether the transgenic lines resistant to CBSV are also resistant to UCBSV. Following a similar procedure, selected transgenic and control scions were grafted onto UCBSV-infected Ebwanateraka rootstocks. Appearance of CBSV symptoms was monitored over three months. CBSD-associated leaf symptoms appeared on control scions at 4 wpg. As in earlier studies [2], [26] UCBSV produced milder symptoms compared to the more virulent CBSV isolate used in the experiments described above. UCBSV accumulated at levels higher than those of CBSV in both rootstocks and scions (Figure 1A, B). Control scions consistently accumulated high UCBSV loads when grafted on UCBSV-infected rootstocks. Scions from the selected transgenic lines remained symptom-free and virus quantitation demonstrated the absence of UCBSV replication (Figure 1B, Table S4).

### Resistance to CBSV and UCBSV can be Transferred to a Farmer-preferred Landrace Resistant to CMD

Most studies reporting engineered improved traits in cassava have so far been performed in cv. 60444 because it can be transformed more efficiently than other cassava cultivars [27], [29]. However, cv. 60444 is not used by African farmers partly because of its high susceptibility to CMD. Because CBSD and CMD occur concomitantly in several cassava-growing regions [4], [5], mitigation of cassava viral diseases requires combination of both CMD and CBSD resistances. Natural resistance to CMD has been previously identified in the cassava germplasm designated the Tropical Manihot Esculenta series (TME) [8], [19]. CMD-resistant TME landraces and their derivatives have been subsequently distributed and readily adopted by farmers in regions where CMD was endemic as part of the CMD mitigation program [7]. Field surveys and greenhouse experiments have recently provided evidence that many CMD-resistant or tolerant cassava cultivars and landraces currently grown in CBSD-endemic regions are susceptible to viruses causing CBSD [2], [5].

The transformation protocol for the TME 7 cassava landrace (locally referred to as Oko-Iyawo) was optimized using the improved transformation protocol [20], [30]. Multiplication of TME 7 friable embryogenic callus (FEC) in the dark combined with extended regeneration steps following *Agrobacterium*-mediated FEC transformation sufficed to implement a high regeneration frequency of transgenic plantlets with the TME landraces [31]. The optimization step of the transformation protocol was particularly instrumental to ensure the timely production of several independent transgenic TME 7 lines necessary for the evaluation of the hairpin-based strategy. A set of 17 TME 7 plantlets was characterized by Southern blot (Figure S3B) and five independent transgenic lines were selected for analysis of hairpin-derived small RNAs accumulation (Figure S4B). Hairpin-derived small RNAs accumulated to comparable levels in all selected lines. Evaluation of the transgenic TME 7 lines followed the procedure established for the transgenic 60444– Hp lines. Wild-type TME 7 grafted on CBSV-infected rootstocks developed typical CBSV symptoms at 5–6 wpg (Figure 3A). All transgenic scions remained symptom-free. Despite variable CBSV viral loads in the cv. 60444 rootstocks, wild-type TME 7 scions consistently supported CBSV replication levels similar to those of wild-type cv. 60444 scions (Figure 4A) using the top-grafting method. Quantitation of CBSV load confirmed the absence of viral replication in all transgenic scions at 10 wpg (Figure 4A, Table S5). Two transgenic TME 7 lines were tested subsequently for resistance to UCBSV. Wild-type TME 7 scions developed typical UCBSV symptoms at 5–6 wpg when grafted on UCBSV-infected rootstocks (Figure 3B). UCBSV symptoms in TME 7 were milder than those of CBSV (compare Figure 3A and B). None of the transgenic scions displayed UCBSV symptoms. Molecular quantitation of UCBSV confirmed the absence of viral replication in the transgenic scions (Figure 3D, Table S4). Despite the development of milder CBSD symptoms, TME 7 supported higher replication of UCBSV as compared to CBSV (Figure 4A, B).

### CBSV and EACMV Co-inoculation does not Alter Engineered CBSV Resistance in Transgenic Cassava

Viruses develop various types of interactions when co-inoculated in a host plant [32]. CBSD viruses have been previously reported to act synergistically with geminiviruses when co-inoculated in *Nicotiana benthamiana* [6]. Geminiviruses interfere with the host silencing machinery through different mechanisms, such as expression of silencing suppressors [33], [34], alteration of the host transcriptome [35] and saturation of the host silencing machinery with geminivirus-derived small RNAs produced on infection [23]. Therefore it was essential to test whether the hairpin-based CBSD resistance in transgenic cassava is altered by the replication of cassava geminiviruses. TME 7 is reported to be CMD-resistant under field conditions [8], [19], [36]. We selected an EACMV-Ug isolate known to cause severe mosaic symptoms in cassava [37]. When inoculated with EACMV-Ug alone or in combination with CBSV using the stringent top-grafting method, TME 7 scions developed typical mosaic symptoms on the first emerging leaves followed by a recovery phenotype in which CMD-associated symptoms became attenuated.

Selected transgenic TME 7–Hp (TME 7-Hp 11 & TME 7-Hp 12) scions were grafted on cv. 60444 rootstocks co-inoculated with CBSV and EACMV-Ug. Rootstock plants infected with the two viral species developed typical CMD symptoms. Sporadic CBSV-associated symptoms were observed on leaves with reduced CMD-associated symptoms (Figure S6A). CBSV and EACMV-Ug were not mutually exclusive in their natural host as demonstrated by the onset of typical CMD and CBSD-associated symptoms in the scions (Figure S6B). Upon grafting on co-infected rootstocks the TME 7– Hp scions displayed CMD-associated symptoms between 3 and 4 wpg. Interestingly, transgenic scions only developed CMD symptoms whereas wild-type scions displayed both CMD and CBSD-associated symptoms with a predominance of CBSD-associated symptoms. The CBSV load was quantitated in the wild-type scions at 8 wpg in the first fully expanded leaves and RT-qPCR data confirmed that CBSV replication occurred in wild-type scions when associated with EACMV-Ug (Figure 5 A). The CBSV load was noticeably highly variable in the wild-type scions. In TME 7–Hp 11 line, which remained free of CSBD-associated symptom, CBSV could replicate to titers in the range of the lowest viral titers observed in the wild-type scions (Figure 5A). In order to evaluate whether CBSV could replicate in scions recovering from EACMV-Ug infection, the CBSV load was quantitated at 14 wpg in all co-inoculated scions. The CBSV load remained variable in wild-type scions whereas CBSV could not be detected in the transgenic scions (Figure 5 B).

## Discussion

The emergence of new viral diseases based on the rapid evolution of existing viruses is threatening and particularly damaging crop production in many tropical countries [38], [39]. The difficulty encountered in rapidly introgressing virus resistance traits or, in some cases, the lack of durable natural resistance in the germplasm, further limit the options to mitigate viral diseases in important food crops [40]. In recent decades, cassava production in Sub-Saharan Africa has become increasingly constrained by viral diseases. Cassava geminiviruses evolving through recombination and trans-replication are found in most cassava-growing regions [3]. Moreover, CBSVs have re-emerged and expanded partly as a consequence of the CMD mitigation measures [4].

Our objective was to generate cassava plants resistant to both CMD and CBSD. Post-transcriptional gene silencing (PTGS) of the CP gene of *Papaya ringspot virus* in transgenic papaya has so far provided the most durable resistance against a plant RNA virus [41]. Because of its higher virulence [2] we hypothesized that the CBSV-CP sequence will be a better target than the UCBSV sequence to generate broad-spectrum resistance to both virus species causing CBSD. The difference in virulence between CBSV and UCBSV is not yet fully understood. However, virulence is often associated with the production of effective viral silencing suppressors that interfere with the host silencing machinery [42], [43] and consequently could alter PTGS-based engineered resistance.

Similarity between hairpins and their targeted viral sequences has previously been hypothesized to restrict the hairpin-based resistance in transgenic plants to virus species and isolates sharing more than 90% sequence similarity [44]. However, recent studies suggest that the similarity threshold for resistance efficacy might be lower than expected [45], [46]. It has been shown that 21-nucleotide stretches of 100% identity are not essential to silence endogenous genes using virus-induced gene silencing (VIGS) [47]. Recent evidence also suggests that endogenous siRNAs can target mRNAs using imperfectly paired target sites [48]. Those observations indicate that prediction of the silencing potential of the hairpin-derived siRNAs cannot rely solely on their mere similarity with target sequences and should also include additional parameters such as duplex free energy already used to predict microRNA target sites [49], [50]. Our *in silico* analysis using free energy of siRNA-target duplexes showed that our CBSV-CP hairpin could generate many small RNAs potentially targeting virus isolates from both CBSV and UCBSV species.

In our study the transgenic cassava lines expressing hairpin RNA fully homologous to CBSV were consistently resistant to both CBSV and UCBSV using grafting as a stringent inoculation procedure. The precise virus quantitation method [24] was particularly important in confirming that CBSV and UCBSV replication does not occur in the transgenic plants. The present study and work reported elsewhere [15], [26] suggest that virulence as well as careful selection of target sequences should be considered when developing robust broad-spectrum CBSD resistance in cassava.

In addition, our evaluation of engineered CBSD resistance using co-infection of viruses causing CMD and CBSD demonstrated that replicating EACMV-Ug show no or limited interference with the hairpin-based RNAi resistance strategy against CBSD when engineered in a CMD-resistant landrace.

Engineering resistance to the many viral species infecting cassava in the field is a difficult and lengthy task that can be facilitated by exploiting naturally occurring virus resistance in cassava germplasm. Optimization of the transformation protocol for TME cassava landraces [31] facilitated our objective to combine CMD resistance with CBSD resistance in a cassava landrace currently used by African farmers. The stringent virus infection procedure that we used to test the transgenic plants validated the robustness of the engineered CBSD resistance in TME 7. Evaluation of the transgenic plants under field conditions with naturally occurring viral populations will ultimately be necessary to assess their potential for reducing the impact of cassava viral diseases in Africa.

## Supporting Information

Figure S1 **Alignment of selected CBSV-CP and UCBSV-CP sequences (partial CDS).** Alignment was performed using the Clustal W method. (DNASTAR Lasergene). Similarity with the CBSV-TAZ-DES01 is highlighted with black boxes. Sequence used for CBSV-CP hairpin construction is indicated by red arrows.
(TIFF)

Figure S2 **Alignment of UCBSV-[UG:Kab4∶07]-CP and CBSV-[TAZ:DES:01]-CP partial sequences.** Short sequences (21nt) derived from CBSV-[TAZ:DES:01]-CP forming RNA duplexes (free energy<25 kCal moI^−1^) with UCBSV-CP sequence are plotted below the alignment. Detailed information about RNA duplexes is reported in Table S1.
(EPS)

Figure S3 **Southern blot analysis of transgenic cv. 60444 (A) and TME 7 (B) plantlets using the hptII probe.**
(TIF)

Figure S4 **Detection of hairpin-derived small RNAs in selected independent transgenic lines.** Hairpin-derived small RNAs in transgenic cv. 60444 (A) and TME 7 (B) lines were detected with a CBSV-CP specific oligoprobe.
(TIF)

Figure S5 **CBSV and UCBSV quantitation in AR34 rootstocks and cv. 60444 scions.** CBSV quantitation by qPCR in CBSV-infected AR34 roostock plants and corresponding grafted scions from transgenic, wild-type and control lines. Numbers following the cassava line identifiers indicate the biological replicates.
(TIFF)

Figure S6 **Symptoms associated with CBSV and EACMV-Ug co-infection in cassava leaves.** A. Fully expanded leaf of wild-type cv. 60444 rootstock co-inoculated with CBSV and EACMV-Ug. B. Fully expanded leaf of wild-type TME 7 scion grafted on a CBSV and EACMV-Ug co-inoculated rootstock.
(TIF)

Table S1 **Summary table of small RNA duplexes between 21nt CBSV-CP hairpin-derived small RNA and UCBSV-CP target sequence.**
(XLSX)

Table S2 **Summary table of phenotypic and molecular data of the 60444 - Hp transgenic scions.** Transgenic 60444-Hp scions were grafted on CBSV-infected cv. 60444 rootstocks.
(DOCX)

Table S3 **Summary table of phenotypic and molecular data of the 60444 - Hp transgenic scions**. Transgenic 60444-Hp scions were grafted on CBSV-infected AR34 rootstocks.
(DOCX)

Table S4 **Summary table of phenotypic and molecular data of the 60444 - Hp and TME 7 - Hp transgenic** **scions.** Transgenic 60444-Hp and TME 7-Hp scions grafted on UCBSV-infected Ebwanateraka rootstocks.
(DOCX)

Table S5 **Summary table of phenotypic and molecular data of the TME 7 - Hp transgenic scions.** Transgenic TME 7-Hp scions were grafted on CBSV-infected AR34 rootstocks.
(DOCX)
