# Supplementary figures and images for: Exploiting the Combination of Natural and Genetically Engineered Resistance to Cassava Mosaic and Cassava Brown Streak Viruses Impacting Cassava Production in Africa
Source: PLoS One. 2012 Sep 25;7(9):e45277. doi: 10.1371/journal.pone.0045277 (PMC3458115; doi:10.1371/journal.pone.0045277)

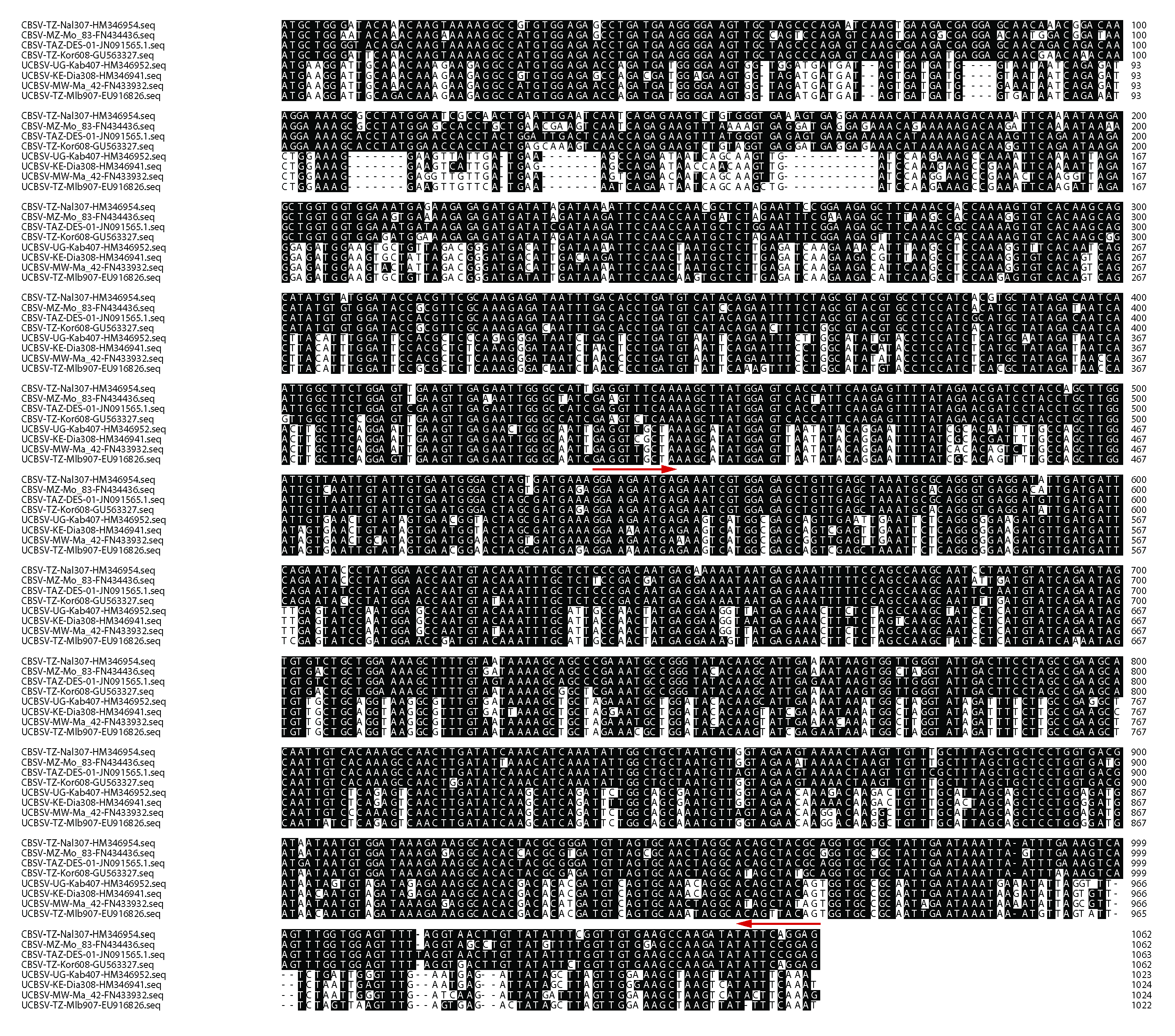

Supplement: Figure S1 — Alignment of selected CBSV-CP and UCBSV-CP sequences (partial CDS). Alignment was performed using the Clustal W method. (DNASTAR Lasergene). Similarity with the CBSV-TAZ-DES01 is highlighted with black boxes. Sequence used for CBSV-CP hairpin construction is indicated by red arrows. (TIFF) [file pone.0045277.s001.tiff]

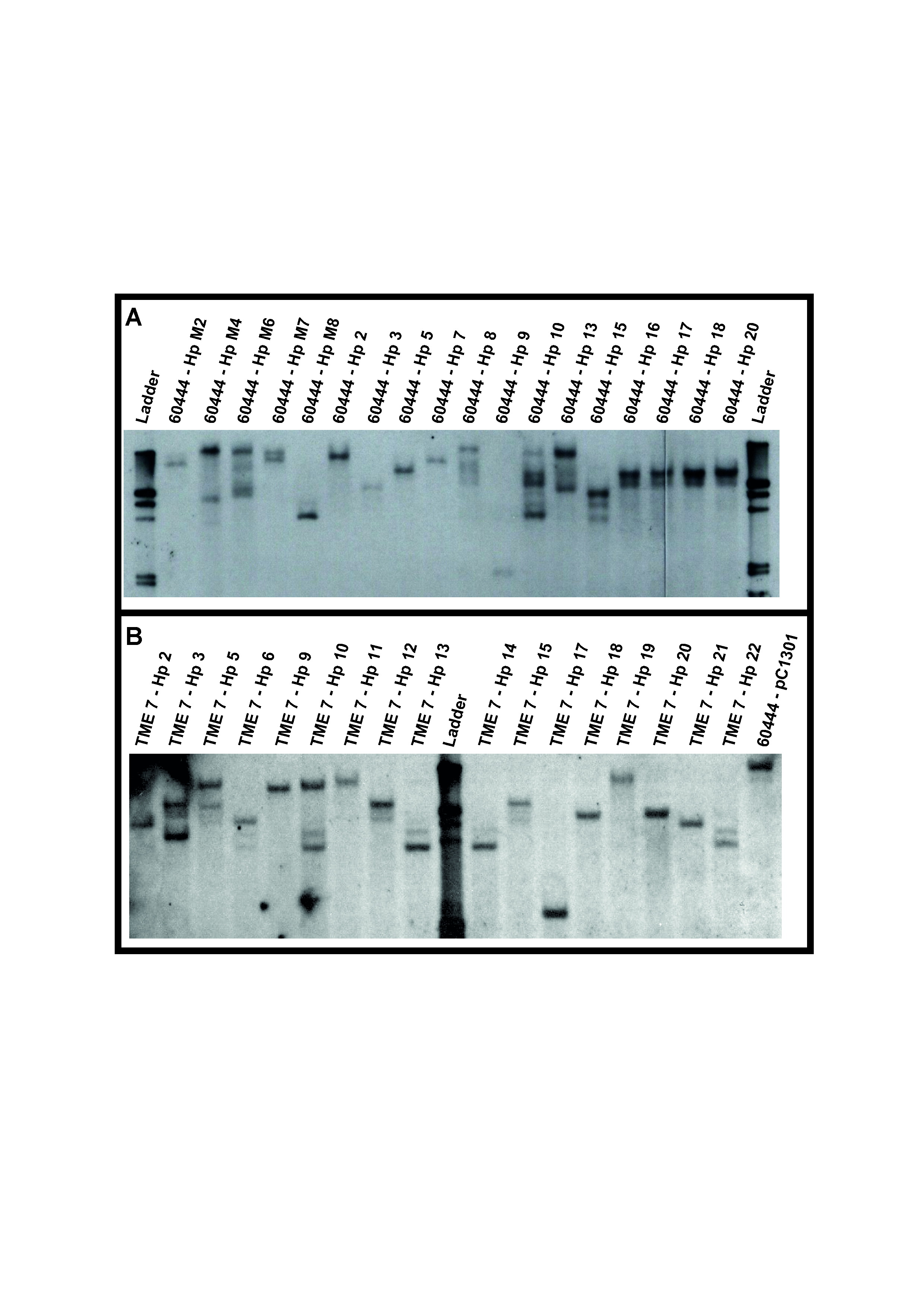

Supplement: Figure S3 — Southern blot analysis of transgenic cv. 60444 (A) and TME 7 (B) plantlets using the hptII probe. (TIF) [file pone.0045277.s003.tif]

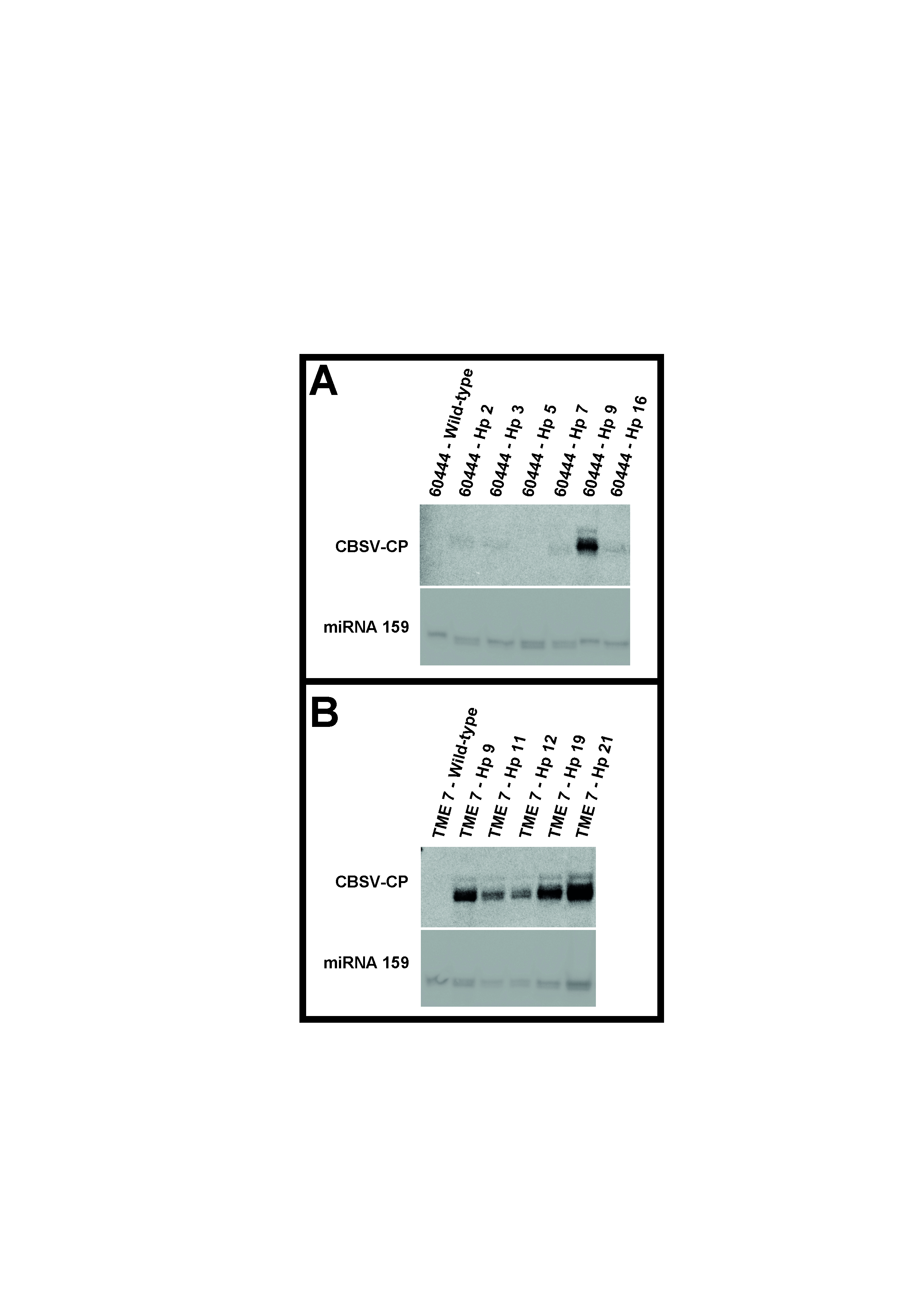

Supplement: Figure S4 — Detection of hairpin-derived small RNAs in selected independent transgenic lines. Hairpin-derived small RNAs in transgenic cv. 60444 (A) and TME 7 (B) lines were detected with a CBSV-CP specific oligoprobe. (TIF) [file pone.0045277.s004.tif]

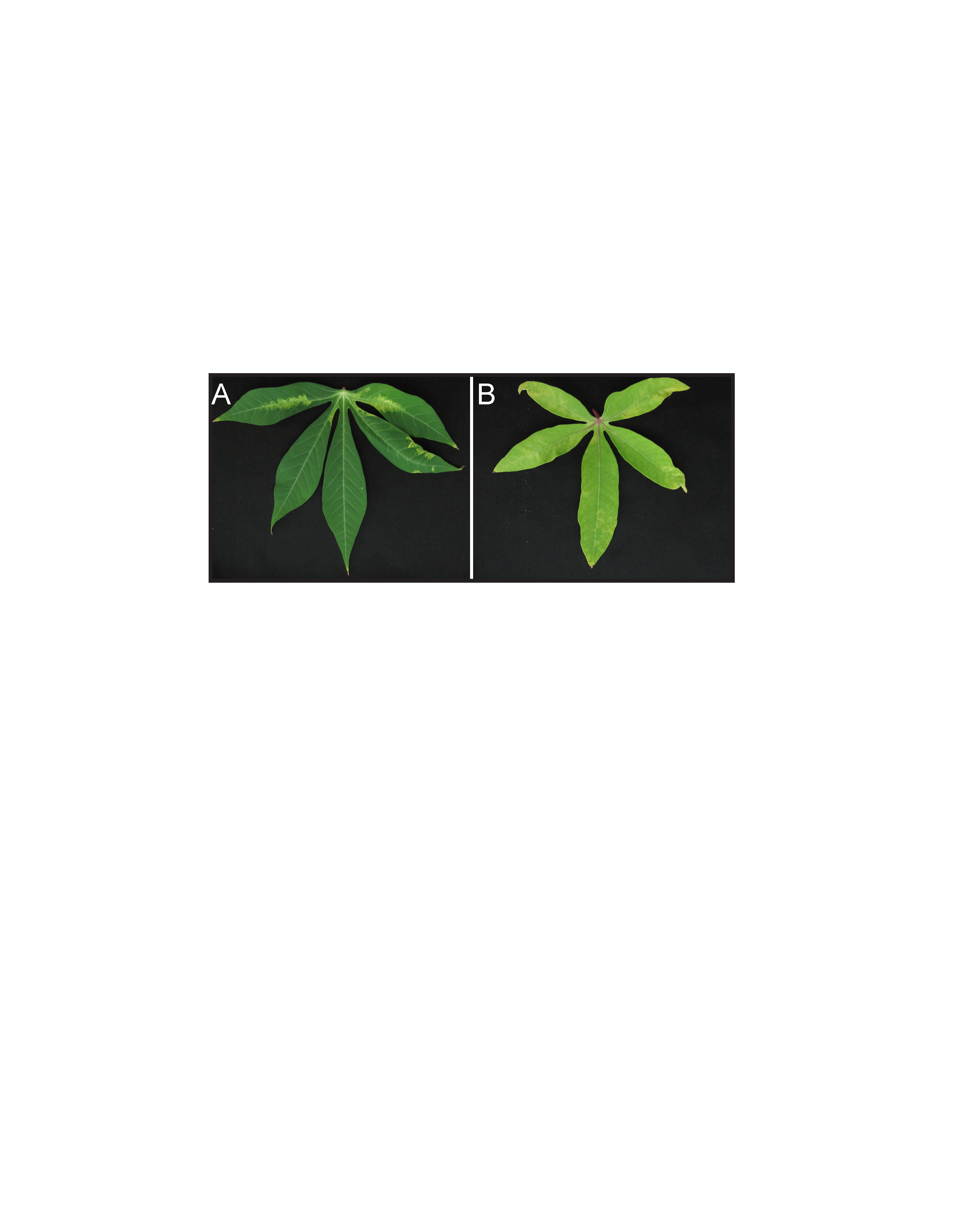

Supplement: Figure S6 — Symptoms associated with CBSV and EACMV-Ug co-infection in cassava leaves. A. Fully expanded leaf of wild-type cv. 60444 rootstock co-inoculated with CBSV and EACMV-Ug. B. Fully expanded leaf of wild-type TME 7 scion grafted on a CBSV and EACMV-Ug co-inoculated rootstock. (TIF) [file pone.0045277.s006.tif]
