# Supplementary material for: Exploiting the Combination of Natural and Genetically Engineered Resistance to Cassava Mosaic and Cassava Brown Streak Viruses Impacting Cassava Production in Africa
Source: PLoS One. 2012 Sep 25;7(9):e45277. doi: 10.1371/journal.pone.0045277 (PMC3458115; doi:10.1371/journal.pone.0045277)
Supplement: Table S2 — Summary table of phenotypic and molecular data of the 60444 - Hp transgenic scions. Transgenic 60444-Hp scions were grafted on CBSV-infected cv. 60444 rootstocks. (DOCX) [file pone.0045277.s008.docx]

**Table S2.** **Summary table of phenotypic and molecular data of the 60444 - Hp transgenic scions.** Transgenic 60444-Hp scions were grafted on CBSV-infected cv. 60444 rootstocks.

TABLE S2

| **Scion identity** | **Rootstock**  **Identity** | **Virus species** | **Number of grafts** | **Symptomatic scions** | **Virus presence in scion (RT-qPCR)** |
| --- | --- | --- | --- | --- | --- |
| 60444 – Wild-type | cv. 60444 | CBSV | 4 | 4 | 4 |
| 60444 – Cambia | cv. 60444 | CBSV | 3 | 3 | 3 |
| 60444 – Hp 2 | cv. 60444 | CBSV | 4 | 0 | 0 |
| 60444 – Hp 3 | cv. 60444 | CBSV | 4 | 0 | 0 |
| 60444 – Hp 5 | cv. 60444 | CBSV | 3 | 3 | 3 |
| 60444 – Hp 7 | cv. 60444 | CBSV | 3 | 0 | 0 |
| 60444 – Hp 9 | cv. 60444 | CBSV | 4 | 0 | 0 |
| 60444 – Hp 16 | cv. 60444 | CBSV | 4 | 4 | 4 |
