# Supplementary material for: Exploiting the Combination of Natural and Genetically Engineered Resistance to Cassava Mosaic and Cassava Brown Streak Viruses Impacting Cassava Production in Africa
Source: PLoS One. 2012 Sep 25;7(9):e45277. doi: 10.1371/journal.pone.0045277 (PMC3458115; doi:10.1371/journal.pone.0045277)
Supplement: Table S4 — Summary table of phenotypic and molecular data of the 60444 - Hp and TME 7 - Hp transgenic scions. Transgenic 60444-Hp and TME 7-Hp scions grafted on UCBSV-infected Ebwanateraka rootstocks. (DOCX) [file pone.0045277.s010.docx]

**Table S4.** **Summary table of phenotypic and molecular data of the TME 7 - Hp transgenic scions.** Transgenic TME 7-Hp scions were grafted on CBSV-infected AR34 rootstocks.

| **Scion identity** | **Rootstock**  **Identity** | **Virus species** | **Number of grafts** | **Symptomatic scions** | **Virus presence in scion (RT-qPCR)** |
| --- | --- | --- | --- | --- | --- |

| TME 7 – Wild-type | AR34 | CBSV | 3 | 3 | 3 |
| --- | --- | --- | --- | --- | --- |
| TME 7 – Hp 9 | AR34 | CBSV | 8 | 0 | 0 |
| TME 7 – Hp 11 | AR34 | CBSV | 8 | 0 | 0 |
| TME 7 – Hp 12 | AR34 | CBSV | 7 | 0 | 0 |
| TME 7 – Hp 19 | AR34 | CBSV | 8 | 0 | 0 |
| TME 7 – Hp 21 | AR34 | CBSV | 8 | 0 | 0 |
